# The application of integrating medical humanities education into emergency skill-training scenario simulation teaching

**DOI:** 10.3389/fmed.2025.1561504

**Published:** 2025-03-07

**Authors:** Hongkun Guo, Huan Li, Yongdong Yao, Yiming Li, Yanjing Huang

**Affiliations:** ^1^Department of Trauma Center and Emergency Surgery, The First Affiliated Hospital, Fujian Medical University, Fuzhou, China; ^2^Department of Infectious, Fujian Provincial Geriatric Hospital, Fuzhou, China; ^3^Department of Emergency, The First Affiliated Hospital, Fujian Medical University, Fuzhou, China; ^4^Department of Emergency, National Regional Medical Center, Binhai Campus of the First Affiliated Hospital, Fujian Medical University, Fuzhou, China

**Keywords:** emergency skill-training, scenario simulation teaching, medical humanities education, communication skills training, curriculum integration, experiential learning

## Abstract

**Objective:**

To explore the value of integrating medical humanities education into emergency skill training scenario simulation teaching.

**Method:**

69 first-year professional master’s students studying at Fujian Medical University(China) were selected as research subjects. They were randomly divided into control (*n* = 39) and observation (*n* = 40) groups. All students received emergency skills training. The control group adopted the scenario simulation teaching method, while the observation group integrated medical humanities education based on the control group. Assessment scores and satisfaction with the teaching mode were compared between the two groups.

**Results:**

The observation group outperformed the control group in practical, theoretical, and comprehensive grades, and were more satisfied with the teaching mode, with both differences being statistically significant (*p* < 0.05).

**Conclusion:**

Incorporating medical humanities education into emergency training simulations can enhance teaching quality, boost students’ ethical literacy, and improve teaching satisfaction, making it worthy of widespread application.

## 1 Introduction

Medical education is a continuous and lifelong process, and postgraduate medical education is an important part of this process (1). With the rapid development of degrees and postgraduate education in China, the training mode of medical postgraduates has undergone tremendous changes after the combination of medical professional degree postgraduate training and resident standardized training (2). However, when postgraduates start clinical practice, their courses are still based on traditional teaching content, and the clinical courses set up by the resident training base are still in the exploratory stage and are still mainly based on spoon-feeding teaching methods. Frequent tests and high-intensity content in traditional teaching courses lead to high stress, increased anxiety, and reduced enthusiasm for learning. Spoonfeeds residents emphasizes the coverage of knowledge and ignores students’ understanding and application, which may affect students’ clinical decision-making ability and empathy cultivation. Although traditional teaching methods are important, they lack interaction and practical application and cannot meet the needs of modern medical education. This has become a major problem that restricts the improvement of the clinical ability of postgraduates after they are incorporated into residents’ standardized training. With the progress and development of medical education and the continuous increase in China’s aging population, the quality requirements for doctors are constantly improving (3). Therefore, the standards for the training of clinical medical professional degree postgraduates have expanded, covering professional ethics, basic medical knowledge, clinical thinking, clinical skills, doctor-patient communication ability, and other contents. However, in the actual training mode, students’ dominant position in teaching is often ignored, and their clinical thinking, professional characteristics, and practical ability are not highlighted, especially in the cultivation of humanistic qualities, which are relatively weak. In the humanistic education of medical postgraduates, training hospitals face many problems and practical difficulties. It cannot meet the requirements of professional and personalized training for professional postgraduates. Around the world, medical schools are incorporating humanities into their curricula to address the need to train compassionate doctors by developing personal attributes such as integrity and respect (4).

Medical humanities education is an educational system aimed at cultivating medical students’ humanistic literacy, ethical concepts, and empathy abilities. Its core lies in integrating humanities and social sciences with the medical profession to cope with the trend of modern medical technology and dehumanization, and to cultivate medical talents who possess both professional skills and humanistic care.

Emergency medicine is a professional discipline with strong practicality and involves multi-disciplinary knowledge. In clinical diagnosis and treatment, it mainly involves the treatment of all kinds of acute and critical diseases and has high requirements for professional operation skills and thinking modes. Therefore, strengthening the training of students’ first-aid skills is of great significance. Scenario simulation teaching mainly refers to the use of various technical methods to simulate the situation of common clinical cases, taking students as the center, and teaching through practical multiple activities. Compared with the traditional teaching mode, the application of scenario simulation teaching helps to mobilize students’ initiative and enthusiasm, combine students’ knowledge with practice, and then help them fully grasp the first-aid process and methods (5, 6). In addition, in the actual teaching stage, we also need to pay attention to medical humanities education, which not only helps students master medical humanities-related knowledge at the theoretical level but also improves their humanistic quality in the process of practice. Traditional “spoon-feeding” methods and siloed clinical courses fail to prepare learners for the complexities of emergency medicine, where technical expertise must coexist with empathy, ethics, and adaptability. Integrating medical humanities into scenario simulation teaching bridges this gap, fostering clinicians who are not only skilled but also compassionate and reflective. This paradigm shift aligns with global trends in healthcare education, which increasingly prioritize holistic competence over rote knowledge. At present, medical humanities education is widely valued and has achieved remarkable results. Based on this, this study analyzes the application value of medical humanities education in scenario simulation teaching of emergency skills training.

## 2 Materials and methods

### 2.1 General material

**Figure 1:**
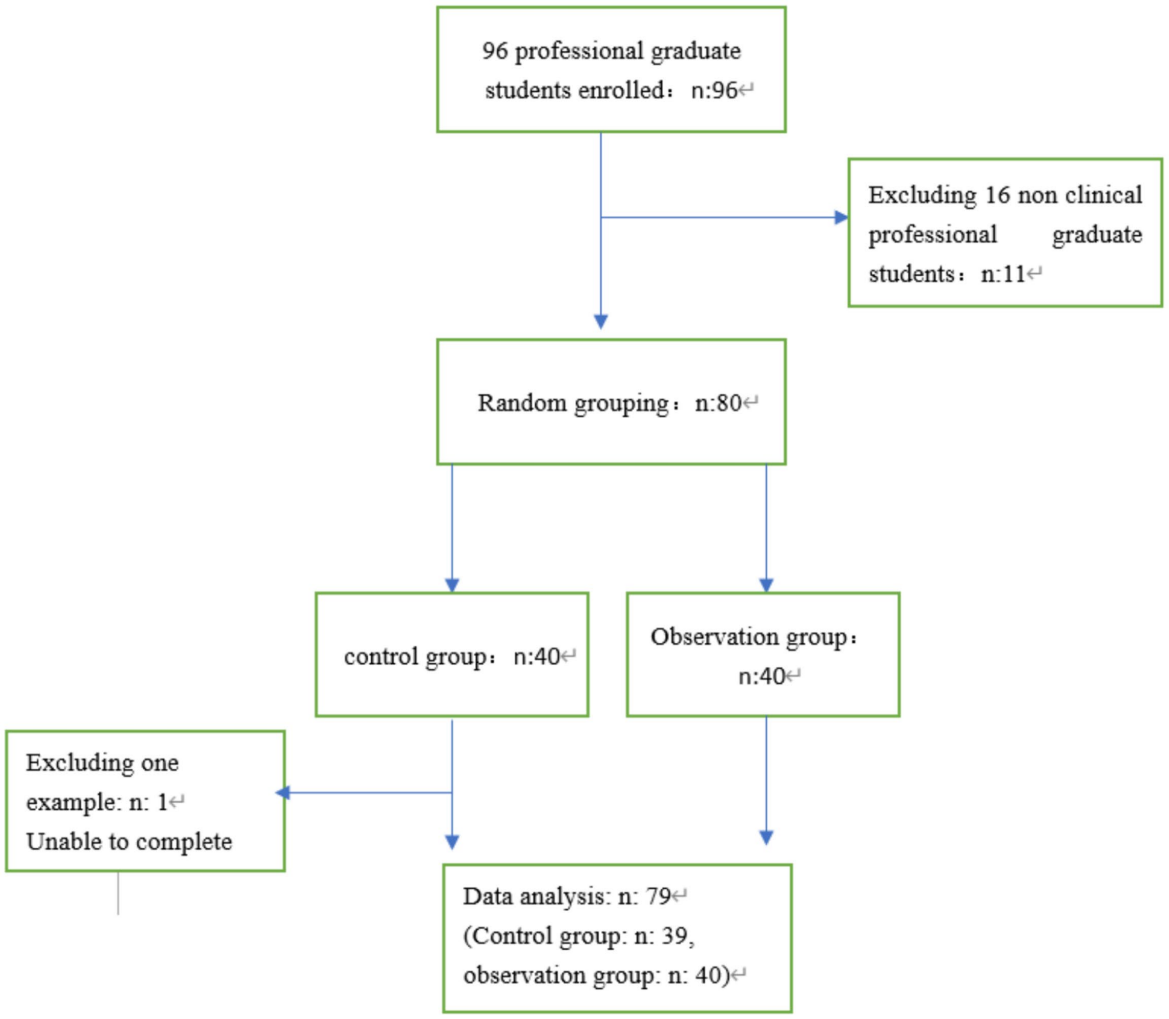
Study flow chat.

96 first-year professional medical postgraduates from Fujian Medical University (China) were selected as research subjects, and 16 non-clinical postgraduates (including medical images, dermatology and venereology, and stomatology) were excluded. They were randomly divided into control and observation groups, with 40 postgraduates in each group. Due to illness, one control group postgraduate was unable to complete the training. 79 postgraduates were included in this study (Figure 1): 39 postgraduates in the control group and 40 postgraduates in the observation group. The number of male and female patients in the control group was 22 and 17, with an average age of (23.52 ± 0.61) years old ranging from 20 to 23 years old; There were 25 males and 15 females in the observation group, with an average age of (23.41 ± 0.77) years old ranging from 20 to 23 years old. There were no significant differences between the two groups in the baseline data of the students (*p* > 0.05), such as category (*p* = 0.926), major (*p* = 0.843), sex (*p* = 0.581), and admission score (*p* = 0.500), which can be used for comparative analysis (Table 1). There was no significant difference between the two groups in terms of student category, major, gender, and admission score (*p* > 0.05), which means that the baseline data of the two groups were consistent and had no difference. The research protocol was approved by the Medical Ethics Committee of Fujian Provincial Geriatric Hospital in China (No. 20240401). Before conducting the study, we obtained written informed consent from all participants. Participants were assured that their participation in the study would not affect their current or future studies. There was no potential harm to participants.

**Table 1:** Comparison of baseline data between control group and observation group.

Items	Categories	Group(%)		Total	χ^2^	*p*
		Control group	Observation group			
Category	Full-time professional master’s degree*	23(58.974)	24(60.000)	47(59.494)	0.009	0.926
	Full-time professional master’s degree (5 + 3)**	16(41.026)	16(40.000)	32(40.506)		
Major	Clinical medicine	16(41.026)	16(40.000)	32(40.506)	4.155	0.843
	Pediatrics	1(2.564)	0(0.000)	1(1.266)		
	General practice	1(2.564)	2(5.000)	3(3.797)		
	Internal medicine	5(12.821)	7(17.500)	12(15.190)		
	Surgery	11(28.205)	13(32.500)	24(30.380)		
	Obstetrics and gynecology	1(2.564)	0(0.000)	1(1.266)		
	Emergency medicine	2(5.128)	1(2.500)	3(3.797)		
	Ophthalmology	1(2.564)	0(0.000)	1(1.266)		
	Neurology	1(2.564)	1(2.500)	2(2.532)		
Gender	Female	17(43.590)	15(37.500)	32(40.506)	0.304	0.581
	Male	22(56.410)	25(62.500)	47(59.494)		
Admission score	Excellent	10(25.641)	6(15.000)	16(20.253)	1.386	0.500
	Fair	5(12.821)	6(15.000)	11(13.924)		
	Good	24(61.538)	28(70.000)	52(65.823)		

*Full-time professional master’s degree: Completed 5 years of undergraduate studies and participated in and passed the national graduate entrance examination after graduation. **Full-time professional master’s degree (5 + 3): From enrollment to graduation, it is a bachelor’s and master’s degree program without passing the examination of national unified master’s degree.

### 2.2 Methods

Both groups received first aid skills training. The control group adopted the scene simulation teaching method (Two groups of students have the same course taught by the same teacher, and the training will be conducted from March to August 2024, with each class lasting 90 min.): first, the teacher imparted theoretical knowledge, and according to the requirements of the syllabus, taught the operation process of cardiopulmonary resuscitation, trauma treatment, poisoning rescue, and other first-aid skills by using PPT, video, etc. At the same time, the students were divided into groups with corresponding scenes. Design corresponding scenarios that emphasize the need for scenarios to be close to real emergency situations (emergency situations before the hospital and doctor-patient conflicts in the emergency room, etc.), while incorporating ethical dilemmas (such as prioritizing treatment decisions when resources are limited). Set different roles, including medical staff, patients, family members, social workers, etc., and require students to handle composite tasks such as doctor-patient communication and team collaboration in the simulation. And design progressive tasks, such as synchronously calming the emotions of family members during cardiopulmonary resuscitation. Therefore, teachers should focus on guidance. After the scenario simulation (About 25 min), teachers should comment on the deficiencies, help students recognize the problems in time, strengthen their understanding of relevant theoretical knowledge, and improve their practical operation ability.

Observation group: Medical humanities education was integrated based on the control group and the main contents were as follows:

(1) Hospital internship. Strengthening students’ awareness of the reverence of life. Emergency department may be the workplace in the future, and emergency department internships are also a key part of medical education. In clinical practice teaching, we should not only pay attention to the teaching of skills, but also to the transmission of humanistic ideas. It is difficult for students to have direct access to the first-aid scene when studying at school, so they do not have a deep understanding of life and death. The situation of emergency patients is complex, and each life may be in danger of death under different circumstances. Teachers should avoid arranging a certain situation in advance and should strengthen the education of students in combination with the situation of the emergency department and the medical humanities theory of professional graduate students in emergency learning. For example, when a traffic accident occurs, students should be taught first-aid skills on-site, and humanistic education should be strengthened to enhance their awareness of the reverence of life. During the study, students were allowed to have close contact with the scene, improve their cognition of life and death, and strengthen their awareness of reverence in life.
(2) Simulate a scene of unsuccessful rescue so that students can treat death objectively. For example, if a patient with sudden heart disease was sent to the emergency department, the doctor failed to save the patient’s life after first aid and needed to go out of the loss by gaining an understanding of the family members through communication. Through this kind of scenario simulation, students can realize the limitations of medicine, cultivate their communication abilities, and improve their humanistic quality (7).
(3) Simulate emergency safety events and cultivate students’ communication and decision-making abilities Natural disasters, large traffic accidents, and public health emergencies are all emergency medical tasks. During scenario simulation teaching, some scenes can be simulated from films and television plays, such as large-scale traffic accidents, in which each student can be guided to participate without designed-in-advance lines and processes. One group pretended to be medical staff, and one group pretended to be patients and family members. The second is to exchange roles to complete teaching. In the process of scenario simulation, students can use humanistic theory reasonably when communicating and making decisions, and there may be problems such as not being calm or hesitant to deal with the tasks. During the teaching process, teachers guide students to summarize on their own and encourage them to critically reflect on technical performance and humanistic dimensions. Divided into the following stages.

I Simulation practice stage: High fidelity simulation scenarios (such as cardiopulmonary resuscitation, team handling of poisoning cases), pause at key nodes, insert 1–2 min of “micro reflection” (e.g., checking team division of labor).
II Instant reporting stage (time allocation: single simulation and reflection ratio: 1:1 to 1:2, for example: 20 min of simulation+15–30 min of reflection): Reflection was anchored in the PEARLS framework (Promoting Excellence and Reflective Learning in Simulation), which systematically guides learners through three phases:

1) Description: Form: Students verbally summarized their actions during the simulation, What clinical steps did I prioritize during the trauma recovery.
2) Analysis: Instructors posed targeted questions to dissect decision-making, such as: “How did the patient’s emotional state (e.g., anxiety, cultural background) influence your communication strategy?”

“What ethical dilemmas arose when balancing procedural efficiency with pain management?”

3) Application: Learners articulated actionable insights for future practice (e.g., “Next time, I will allocate more time to family counseling before initiating invasive procedures”).

III Written reflection stage: Students upload electronic reflection summaries on the Chaoxing learning platform, and teachers provide structured annotations and feedback.

(4) Give full play to the guiding role of teachers. Teachers have a direct impact on their teaching quality. To mobilize students’ subjective initiative, teachers should give full play to their guiding roles. Therefore, the humanistic education of scene simulation first-aid skills training should select teachers with strong professional ability, rich experience, and high humanistic quality to improve teaching quality, strengthen the cultivation of students’ humanistic quality, and make students truly feel humanistic care.
(5) Integrating narrative medicine into teaching. The emergency department is not only an important carrier of emergency medicine, but also an important place in the hospital. It displays stories of death, pain, aging, joy, and gratitude after a successful rescue every day. With the development of humanistic medicine and diversification of patients’ needs and values, the use of narrative medicine can enable emergency medical staff to fully understand and respect patients. Different patients had different characteristics. Instead of using the conventional single mode, patients should receive personalized medical care. Through the integration of narrative medicine in teaching, students can fully consider the personal and family factors of patients and give them an appropriate and intimate diagnosis and treatment, so that patients can feel care, warmth, and dignity.

### 2.3 Teaching assessment

In this study, the improvement of teaching quality is evidenced by advancements in learners’ empathy, moral reasoning, communication efficacy, and clinical processing skills, alongside improvements in students’ academic performance and their satisfaction with the educational experience.

#### 2.3.1 Process assessment

After the first aid skills training and teaching, the performance of postgraduates in the experimental and control groups in the process of skill operation was scored by formative evaluation. The scoring work is completed by a team of clinical physicians with intermediate or higher professional titles, and each evaluation includes at least 2 independent evaluators (such as attending physicians or associate chief physicians). To ensure standardized scoring, all evaluators must receive unified training before the evaluation: (1)Standardized training: Familiarize oneself with the scoring rules of Mini CEX and DOPS through simulated assessments, with a focus on strengthening the consistency of subjective dimensions such as humanistic care and communication skills in scoring; (2) Calibration assessment: Use standard cases for simulated scoring, verify inter rater consistency through intra group correlation coefficient (ICC), and require ICC ≥ 0.8 to participate in formal evaluation; (3) Regular refresher training: Update the interpretation of scoring criteria every quarter to reduce scoring bias caused by differences in clinical experience.

The mini CEX developed by the American Board of Internal Medicine (ABIM) and the DOPS designed by the Royal College of Physicians (RCP) were used to evaluate the clinical skills of residents. The Min CEX was evaluated from seven aspects, including the ability of medical history inquiry, physical examination, humanistic care, clinical judgment, communication skills, organizational effectiveness, and overall performance; Through the 3-level 9-point system, the overall evaluation of students’ ability to care for patients, medical knowledge application, learning and improvement in clinical work, interpersonal and communication skills, professionalism, and professional ability (8, 9). DOPS was assessed in 11 aspects: understanding the indications and contraindications of this skill operation, informing patients in detail and obtaining consent before operation, preparation work before performing clinical operation skills, good aseptic concept, standardized and correct operation procedures, accurate and skilled operation techniques, appropriate assistance seeking, relevant disposal after performing clinical operation skills, communication skills, whether taking into account patients’ feelings and professional quality, and the performance of clinical operation skills. The clinical skill performance and humanistic care professional quality of students were evaluated using a 3-level 9-point system. The Mini-CEX and DOPS scores are 1–3 points for unqualified, 4–6 points for qualified, and 7–9 points for excellent (10).

#### 2.3.2 Performance assessment after training

After the study, the two groups of postgraduate students were evaluated for their knowledge of several common critical illness rescue skills in the emergency room (cardiopulmonary resuscitation, hemostasis bandage, poisoning gastric lavage, fracture fixation, spinal trauma transport, airway opening support, etc.), mainly including theoretical achievement, full score of 50, practical operation achievement, and full score of 50. The comprehensive score equals the theoretical achievement plus practical operation achievement, with a full score of 100.

#### 2.3.3 Satisfaction survey

After the training, the two groups were surveyed using anonymous and independent questionnaires. Comparing the satisfaction of the two groups with the teaching mode, the main evaluation contents include the following aspects: whether it helps to improve the operation skills, whether it helps to improve the learning interest, whether it helps to improve the communication ability, whether it helps to improve the understanding of the disease development process and whether it helps to improve the problem-solving ability. These can be divided into satisfaction and dissatisfaction. Satisfaction = number of satisfied cases/total number of cases×100%. Among them, 69 questionnaires were distributed to the control and observation groups, and 69 were recovered, with a recovery rate of 100%.

### 2.4 Statistics

The data obtained from the study were processed and analyzed by spss22.0, using *X* ± s to represent the measurement data, and using *t*-test, % to represent the count data, and *x^2^* for inspection. *p* < 0.05, indicating that there was a statistically significant difference between the groups.

## 3 Results

### 3.1 The min-CEX scores of the two groups after the first aid skills training and teaching

Table 2 shows that the abilities of the control group and the observation group for medical history inquiry, physical examination and other abilities are significant (*p* < 0.05), which means that there are significant differences among different grouped samples, and the score of the observation group is higher.

**Table 2:** Comparison of min CEX scores between two groups.

	Groups (Mean ± Std. deviation)		*t*	*p*
	Control group (*n* = 39)	Observation group (*n* = 40)		
Medical-history inquiry	5.385 ± 1.067	6.875 ± 0.822	−6.966	0.000***
Physical examination	4.692 ± 0.977	6.475 ± 0.847	−8.67	0.000***
Humanistic care,	4.256 ± 0.850	6.400 ± 0.928	−10.699	0.000***
Clinical judgment,	4.769 ± 0.902	6.400 ± 0.955	−7.797	0.000***
Communication skills	4.641 ± 0.811	6.750 ± 0.776	−11.812	0.000***
Organizational effectiveness	4.667 ± 0.898	6.775 ± 0.832	−10.828	0.000***
Overall performance	4.692 ± 0.863	7.000 ± 0.751	−12.687	0.000***

*p < 0.05; **p < 0.01; ***p < 0.001.

### 3.2 Comparison of DOPS scores between the two groups after first aid skills training and teaching

Table 3 shows that the DOPS scores of the two groups are significantly different (*p* < 0.05), and the score of the observation group is higher.

**Table 3:** Comparison of DOPS scores between two groups.

	Groups (Mean ± Std. deviation)		*t*	*p*
	Control group (*n* = 39)	Observation group (*n* = 40)		
Understanding of the indications and contraindications of this skill operation	4.692 ± 0.893	6.750 ± 0.927	−10.045	*p* < 0.05
Detail informing and obtaining consent before operation.	4.641 ± 0.873	6.875 ± 0.911	−11.122	*p* < 0.05
Preparation work before performing clinical operation skills	4.769 ± 0.706	6.750 ± 0.809	−11.588	*p* < 0.05
Good aseptic concept	4.718 ± 0.944	6.775 ± 0.862	−10.116	*p* < 0.05
Standardized and correct operation procedures	4.846 ± 0.961	6.700 ± 0.992	−8.433	*p* < 0.05
Accurate and skilled operation techniques	4.615 ± 0.990	6.775 ± 0.832	−10.51	*p* < 0.05
Seeking assistance appropriately	4.821 ± 0.854	6.650 ± 0.864	−9.462	*p* < 0.05
Relevant disposal after performance	4.410 ± 0.850	6.600 ± 0.810	−11.725	*p* < 0.05
Communication skills	4.359 ± 0.873	6.750 ± 0.742	−13.123	*p* < 0.05
Concept of injury caring	4.256 ± 1.069	6.850 ± 0.893	−11.714	*p* < 0.05
Overall performance	4.821 ± 0.970	7.050 ± 0.846	−10.897	*p* < 0.05

### 3.3 Comparison of examination results of two groups

The observation group had significantly higher practical operation, theoretical, and comprehensive scores than the control group (*p* < 0.05; Table 4).

**Table 4:** Comparison of assessment scores between two groups (*x* ± s, points).

Group	Numbers	Theoretical scores	Operation scores	Comprehensive scores
Control group	39	38.18 ± 2.97	37.92 ± 2.08	76.10 ± 4.38
Obsercation group	40	43.68 ± 2.48	42.03 ± 2.34	83.90 ± 3.75
t		−6.368	−8.230	−8.508
p		<0.05	<0.05	<0.05

### 3.4 Comparison of satisfaction levels between two groups

The satisfaction of the observation group with the teaching mode was significantly higher than that of the control group (*p* < 0.05; Table 5).

**Table 5:** Comparison of teaching mode satisfaction between two groups [n (%)].

	Groups (Mean ± Std. deviation)		x^2^	*p*
	Control group(*n* = 39)	Observation group(*n* = 40)		
Whether it helps to improve the operation skills	29(74.36)	37(92.50)	4.727	*p* < 0.05
Whether it helps to improve the learning interest	25(64.10)	35(87.50)	5.918	*p* < 0.05
Whether it helps to improve the communication ability	25(64.10)	36(90.00)	7.528	*p* < 0.05
Whether it helps to improve the understanding of the disease development process	26(66.67)	35(87.50)	4.872	*p* < 0.05
Whether it helps to improve the problem-solving ability.	27(69.23)	36(90.00)	5.274	*p* < 0.05

## 4 Discussion

Medical humanities is an interdisciplinary field that includes anthropology, sociology, Ethics, psychology, literature, art, and history, as they integrate medical practice (11). Its theoretical framework includes four dimensions: instrumentality (enhancing clinical skills such as communication and empathy), internalization (balancing science and human education), criticality (reflecting on power relations in medical practice), and epistemology (exploring the cultural and social construction of medical knowledge). For example, narrative medicine cultivates empathy by listening to patients’ stories, while medical ethics courses strengthen professional ethics (12, 13). Emergency medicine is a major component of clinical practice. As a specialized discipline, it requires the use of minimal data and the shortest time to save lives and alleviate pain. It has characteristics such as criticality and randomness and involves a wide range of professional knowledge and operational skills (5, 14). Students will be healthcare workers in the future. Medical humanities education determines the comprehensive quality. Only with the spirit of humanistic care can they value the safety of patients’ lives. When cultivating emergency medical personnel, it is necessary to consider humanities as a key skill, strengthen their professional ethics, and strengthen their emergency care. Research has shown that both hospital and school teaching require active promotion of medical humanities education, with a focus on cultivating students’ humanistic qualities (4, 15, 16).

Medical practice should include humanistic care, so medical technology and medical humanities are closely related, and the two promote and complement each other. Although medical technology is developing rapidly, humanistic care in the medical profession is becoming increasingly weak. There is a clear lack of humanistic care in medical development and practice, as well as a lack of cultivation of humanistic spiritual literacy in medical education. For a qualified doctor, it is not only necessary to have a solid theoretical knowledge system and master professional operational skills but also to have noble humanistic literacy (17–19). The integration of empathy and compassion into medical curricula is critical to address the growing concern of dehumanization in clinical practice, particularly in high-stakes emergency settings. Studies reveal significant gaps in current training: only 3.1% of medical students demonstrate familiarity with narrative medicine, a cornerstone of empathy development, and 63.1% lack interest in further learning despite recognizing its clinical value (20). Furthermore, longitudinal research highlights empathy erosion during traditional clinical training, especially in programs with delayed patient interaction, whereas curricula emphasizing early patient engagement and reflective practices show stabilized or improved empathy levels (21). Compared to other medical activities, emergency medicine places higher demands on the humanistic literacy of medical personnel.

Richard Shin, Kent Li, et al. found through multi center emergency resident physician simulation training (such as cardiovascular emergency team collaboration) that participants’ teamwork and communication skills significantly improved, which is consistent with the goal of “doctor-patient communication in emergency settings” in this study, but it focuses more on technical skills rather than internalization of humanistic theories (22). Javier González-Blázquez et al. emphasized the impact of bioethical education on medical decision-making, pointing out that problem-based learning (PBL) (23) can enhance students’ ethical reflection ability, but its research scenarios are mostly traditional classrooms and have not been combined with high fidelity simulation environments. This study combines high fidelity scenario simulation with ethical practice to demonstrate the unique promoting effect of scenario simulation on ethical practice ability. This study combines the current characteristics of emergency medical education and introduces scenario simulation teaching, narrative medicine, and medical humanities education into the process of emergency skills training. The research showed that the observation group’s practical skills operation score (42.03 ± 2.34), theoretical score (43.68 ± 2.48), and comprehensive score (83.90 ± 3.75) were significantly higher than those of the control group (37.92 ± 2.08, 38.18 ± 2.97, 76.10 ± 4.38, respectively; *p* < 0.05). The patient situation in the emergency department has the characteristics of rapid disease development, hidden conditions, and multi-disciplinary knowledge. In the traditional teaching mode, when students acquire emergency rescue knowledge, they may have only partial understanding and theoretical difficulties. This study introduces scenario simulation teaching and medical humanities education, which are more conducive to students’ understanding and mastery of the causes and pathogenesis of diseases. For example, in patients with acute myocardial infarction, the symptoms may only be “chest tightness” when they come to the emergency room, but during the treatment process, malignant arrhythmias can occur at any time, leading to sudden cardiac and respiratory arrest. Special simulations of successful and unsuccessful rescue scenarios and intuitive learning of such patients in the emergency room can raise questions and respect for life about why patients suddenly experience cardiac and respiratory arrest during the treatment process. Be more proactive in asking questions and searching for relevant literature after class to understand the causes and processes of diseases and understand why effective cardiopulmonary resuscitation can improve the success rate of patient rescue. This will make the mastery of theoretical knowledge more detailed and solid, and pay more attention to details and standardization in the process of skilled operation.

In this study, the Min CEX scores of the observation group showed that abilities in medical history inquiry (6.875 ± 0.822, *p* < 0.05), physical examination (6.475 ± 0.847, *p* < 0.05), humanistic care (6.400 ± 0.928, *p* < 0.05), clinical judgment (6.400 ± 0.955, *p* < 0.05), communication skills (6.750 ± 0.776, *p* < 0.05), organizational efficiency (6.775 ± 0.832, *p* < 0.05), and overall performance (7.000 ± 0.751, *p* < 0.05) were all higher than those of the control group (5.385 ± 1.067, 4.692 ± 0.977, 4.256 ± 0.850, 4.769 ± 0.902, 4.641 ± 0.811, 4.667 ± 0.898, 4.692 ± 0.863, respectively). In the DOSP scores, the observation group had a clear understanding of the indications and contraindications for this skill operation (6.750 ± 0.927, *p* < 0.05), informed patients and obtained consent before the operation (6.875 ± 0.911, *p* < 0.05), familiarized themselves with the operation preparation (6.750 ± 0.809, *p* < 0.05), good aseptic concept (6.775 ± 0.862, *p* < 0.05), standardized and correct operation procedures (6.700 ± 0.992, *p* < 0.05), and accurate operation techniques proficiency (6.775 ± 0.832, *p* < 0.05), seeking assistance appropriately (6.650 ± 0.864, *p* < 0.05), post-operative processing (6.600 ± 0.810, *p* < 0.05), communication skills (6.750 ± 0.742, *p* < 0.05), concept of injury caring (6.850 ± 0.893, *p* < 0.05), overall performance (7.050 ± 0.846, *p* < 0.05), and other aspects were all higher than the control group (4.692 ± 0.893, 4.641 ± 0.873, 4.769 ± 0.706, 4.718 ± 0.944, 4.846 ± 0.961, 4.615 ± 0.990, 4.821 ± 0.854, 4.410 ± 0.850, 4.359 ± 0.873, 4.256 ± 1.069, 4.821 ± 0.970, respectively). In the process of practical skill learning, through role exchange and the integration of narrative medicine in teaching, students can better understand the anxiety of patients and their families in an empathetic way. During the operation process, they paid more attention to some details and humanistic care during the first aid process. They can fully consider the personal and family factors of patients, provide them with appropriate and considerate diagnosis and treatment services, and enable them to feel care, warmth, and dignity. Owing to the mastery of theoretical knowledge and understanding of disease development, it is beneficial for students to master better communication skills, resulting in significant improvements in practical operations, theoretical scores, and comprehensive scores. This indicates that the integration of medical humanities education into emergency skills training scenario simulation teaching has a definite effect, which helps improve teaching quality and students’ comprehensive abilities. However, at the same time, in this study, the observation group students showed a significant improvement in various aspects of learning. However, except for the overall performance score, which is in the range of 7–9, which is considered excellent, the other aspects of the content are still in the range of 4–6, which is considered qualified. Further improvement is needed in the future of teaching research to strengthen interaction and adaptation with students.

Medical humanities education is not an accessory to skills, but a value coordinate system that shapes clinical decision-making. In emergency simulation teaching, humanistic literacy is achieved through contextualized embedding rather than skill based segmentation, such as synchronously training ‘technical accuracy’ and ‘patient dignity maintenance’ in trauma assessment. In scenario simulation teaching, the teaching methods of scenario simulation are optimized by integrating medical humanities education. Medical humanities education was integrated in terms of scenario design, creation, and performance. By using methods such as unsuccessful rescue scenario simulation and sudden safety incidents’ simulation, students’ enthusiasm is mobilized, and their humanistic literacy is further improved. The particularity of medical care determines the essence of humanistic care, and the object of medical services is the human being. It is not only necessary to pay attention to diseases but also to the individual patient, understand their needs, and fully reflect the humanitarian and humanistic care of medical care. In the process of emergency skills training, humanistic education should be actively integrated, and continuous innovation should be achieved to endow humanistic education with new connotations to cultivate high-quality professional talent and meet the talent needs of clinical emergency rescue. Medical humanities education plays an important role in improving the professional competencies of medical personnel. Integrating medical humanities education into teaching can help to cultivate students’ comprehensive qualities. This study conducted a follow-up survey on the satisfaction of two groups of students in the teaching mode after teaching, and found that the observation group had several aspects, including whether it helped improve operational skills (*p* < 0.05), whether it helped to increase learning interest (*p* < 0.05), whether it helped to improve communication skills (*p* < 0.05), whether it helped to improve understanding of the disease development process (*p* < 0.05), and whether it helped to improve problem-solving ability (*p* < 0.05). Satisfaction with the teaching mode was significantly higher than that of the control group. Integrating humanistic education into simulation teaching of emergency skills training scenarios has a definite effect and can improve student satisfaction.

## 5 Insufficient

There still remains shortcomings in this study has some limitations. (1) Although non-medical clinical majors (such as imaging and dentistry) were excluded from the 69 first-year clinical postgraduate students who participated in this teaching research, their own mastery of emergency medical knowledge, emergency skills, and medical humanities knowledge was also different, which affected the evaluation of the two teaching modes to some extent. (2) This study only involved a few emergency operations, such as cardiopulmonary resuscitation, trauma management, and poisoning rescue. The content still needs to be enriched, and further research should be conducted to increase the teaching and medical humanities content of other emergency skill operations. (3) The interaction between teachers and students still needs to be strengthened, and the exchange of roles between teachers and students can be arranged. Students can collect information and share their medical humanities knowledge about the first-aid process, thereby enhancing their understanding of the essence of humanistic care in the medical process. (4) Potential confounding factors such as lack of unified mentor training in the early stage, asynchronous student training times, and data acquisition methods may affect the results. Unified mentor training or optimized research design for future research to improve comparability of results.

## 6 Conclusion

In summary, the integration of medical humanities education into emergency skills training scenario simulation teaching has extremely high application value, which helps improve teaching quality and is worthy of promotion and application.

## Data Availability

The original contributions presented in the study are included in the article/supplementary material, further inquiries can be directed to the corresponding author.
